# Glycogen Synthase Kinase 3 Is Essential for Intestinal Cell Niche and Digestive Function

**DOI:** 10.3390/biology14111551

**Published:** 2025-11-05

**Authors:** Minggang Yang, Xiaohui Li, Jiajia Zhan, Rui Pan, Ziye Yang, Mengsha Zhou, Lei Ma, Chenfeng Liu

**Affiliations:** 1 School of Life Science, Anhui Medical University, Hefei 230032, China; yang_minggang1001@163.com (M.Y.); jjzhan2025@163.com (J.Z.); ruipan2025@163.com (R.P.); ahmuziye@163.com (Z.Y.); 2 State Key Laboratory of Cellular Stress Biology, School of Life Sciences, Faculty of Medicine and Life Sciences, Xiamen University, Xiamen 361102, China; zhoumsh28@163.com; 3 Department of Geriatrics, The 960Th Hospital of PLA, Jinan 250031, China; xiaohuili070801@163.com; 4 Tianjin Blood Center, Tianjin 300110, China

**Keywords:** glycogen synthase kinase 3, intestinal cell niche, Paneth cells, digestive function, β-catenin

## Abstract

**Simple Summary:**

Glycogen synthase kinase 3 (GSK3) contains two isoforms, GSK3α and GSK3β, which are key negative regulators of the Wnt/β-catenin signaling pathway. This study focuses on investigating the role of GSK3 in intestinal function. We found that GSK3α and GSK3β exhibit functional redundancy in the intestine. However, complete loss of GSK3 resulted in lethality in mice, accompanied by disruption of the intestinal cellular niche, aberrant proliferation and mislocalization of stem cells and Paneth cells, as well as impaired intestinal absorption and motility. Despite that both GSK3 deficiency and APC mutation led to upregulated β-catenin expression, the intestinal phenotypes of GSK3-deficient mice were different from Apc^Min/+^ mice. Notably, deletion of β-catenin partially rescued the hyperproliferation and mislocalization of Paneth cells and contributed to the restoration of the intestinal niche and function. Our findings indicate the essential role of GSK3 in maintaining intestinal homeostasis and reveal the dual function of the GSK3/β-catenin axis in regulating intestinal stem cell development and absorptive function.

**Abstract:**

WNT/β-catenin signaling is essential for intestinal stem cell development and self-renewal, while its dysregulation can drive tumorigenesis. GSK3, a key negative regulator of β-catenin, in intestinal homeostasis remains incompletely understood. In this study, we investigated the role of GSK3 in intestinal development, niche maintenance, and physiological function. Unlike Apc^Min/+^ mice that developed intestinal polyps, neither GSK3α nor GSK3β deficiency disrupted intestinal homeostasis. However, complete GSK3 deletion (DKO) resulted in perinatal lethality, characterized by disturbed crypt–villus architecture, Paneth cell redistribution, and villus elongation. GSK3 deficiency disrupted the intestinal niche, leading to expanded and mislocalized stem cells and Paneth cells, along with reduced tuft and enteroendocrine cells. These alterations impaired nutrient absorption and gut motility. Mechanistically, β-catenin-positive cells were significantly increased following GSK3 deletion. Genetic ablation of β-catenin under GSK3-deficient conditions reduced stem and Paneth cell populations while restoring tuft and enteroendocrine cells, thereby ameliorating niche abnormalities and improving absorptive and peristaltic functions. This study indicates the essential role of GSK3/β-catenin signaling in maintaining intestinal niche integrity and digestive physiology, highlighting potential therapeutic targets for intestinal and digestive disorders.

## 1. Introduction

The intestine is the primary digestive organ, where villous epithelial cells break down carbohydrates, proteins, and fats into absorbable small molecules to maintain energy and nutrient balance [1]. Additionally, the intestinal lumen harbors diverse immune cells and microbiota, which collectively preserve mucosal barrier integrity and synthesize essential vitamins and short-chain fatty acids [2]. Furthermore, the intestine exerts endocrine functions by releasing hormones, such as serotonin and cholecystokinin, thereby regulating metabolic and neural signaling [3]. These multifaceted functions rely on precise coordination among distinct intestinal cell types: Lgr5^+^ (leucine-rich repeat-containing G-protein coupled receptor 5) intestinal stem cells drive epithelial renewal; Paneth cells secrete antimicrobial peptides and provide Wnt signals to support stem cell proliferation and differentiation; enterocytes enable nutrient absorption via microvilli; goblet cells produce mucins to protect the epithelial surface; and enteroendocrine cells release hormones that modulate gastrointestinal motility [4]. Although these cell populations interact to maintain niche stability through controlled proliferation, spatial organization, and apoptosis, the regulatory mechanisms underlying this dynamic equilibrium remain incompletely characterized.

Recent studies have elucidated how multiple signaling pathways regulate intestinal stem cell development and homeostasis. The Wnt/β-catenin pathway maintains stemness by activating downstream targets such as c-Myc and cyclin D1 [5], while the notch pathway regulates cell fate decisions through Delta-like ligands to inhibit differentiation and promote proliferation [6]. The BMP (bone morphogenetic protein) pathway balances stem cell dynamics via Smad (suppressor of mother against decapentaplegic)-mediated signaling [7,8]. During injury repair in rabbits, the TGFβ (transforming growth factor beta)/Smad and Hippo/Yap (yes-associated protein)/Taz (transcriptional co-activator with PDZ-binding motif) pathways act synergistically to activate regenerative stem cells and promote crypt regeneration, thereby ensuring epithelial homeostasis [9]. Notably, the Wnt/β-catenin pathway exhibits a “double-edged sword” characteristic: it is essential for embryonic development and organogenesis, yet its aberrant activation drives tumorigenesis. In Apc^Min/+^ (adenomatous polyposis coli) models, for instance, stabilized β-catenin upregulates c-Myc and cyclin D1, leading to stem cell hyperproliferation and malignant transformation [10]. Similarly, dysregulation of the hippo pathway induces nuclear translocation of Yap/Taz, which is associated with aggressive colorectal cancers [11]. These findings indicate the dual roles of Wnt/β-catenin and other signals in intestinal biology and oncogenesis, highlighting the necessity for precise regulation of this pathway and exploring its upstream inhibitors to maintain intestinal homeostasis and prevent cancer progression.

GSK3, an evolutionarily conserved serine/threonine kinase, comprises two isoforms, GSK3α and GSK3β. Unlike most kinases that require activation via phosphorylation, GSK3 is constitutively active after expression, allowing it to critically regulate processes such as metabolism, neurodevelopment, and tumorigenesis [12]. Although structurally homologous, GSK3α and GSK3β exert distinct biological functions [13]. Studies show that GSK3β deletion leads to embryonic lethality [14], whereas GSK3α-deficient mice develop normally after birth, indicating that GSK3β plays a more dominant role in physiological and pathological contexts [15]. GSK3 is well established as a negative regulator of β-catenin, promoting its ubiquitination-mediated degradation [16], and as a key modulator of Wnt/β-catenin signaling, which may be essential for intestinal stem cell maintenance and epithelial homeostasis. Previous work has demonstrated that GSK3 affects the expression of tight junction and adhesion junction proteins, including occludin, claudin-1, and E-cadherin, thereby contributing to intestinal epithelial barrier function [17]. However, it remains unclear whether GSK3 deficiency leads to functional impairments in intestinal stem cells, accelerates intestinal tumor progression, or compromises intestinal absorption capacity.

Our study employed Villin-Cre and Villin-ERT2-Cre mouse models to induce intestine-specific deletion of GSK3, enabling the investigation of its role in intestinal cellular niche organization, nutrient absorption, and peristaltic function, using immunofluorescence and flow cytometry to analyze cellular composition. The results provide a promising therapeutic target for gastrointestinal pathologies and digestive disorders.

## 2. Materials and Methods

### 2.1. Mice

The *Gsk3α*^fl/fl^, *Gsk3β*^fl/fl^, and *Ctnnb1*^fl/fl^ (Jax stock 022775) mouse strains have been previously described [18] and were kindly provided by Professor Wen-Hsien Liu at Xiamen University. The Villin-CreERT2 line was generously shared by Professor Mo Wei at Xiamen University, while *Apc*^Min/+^ (Jax stock 002020) and Villin-cre (Jax stock 004586) mice were originally obtained from the Jackson Laboratory. All animals were maintained on a C57BL/6 genetic background and housed under specific pathogen-free conditions in the Experimental Animal Center of Xiamen University, with a controlled 12 h light/dark cycle. All experimental procedures were approved by the Animal Care and Use Committee of Xiamen University (Ethics Approval No. XMULAC20170323; approval date: 23 March 2017) and conducted in accordance with institutional animal welfare guidelines and IACUC policies.

All mouse experiments in this study were conducted in compliance with the 3R principles (replacement, reduction, and refinement). Mice that required euthanasia post-intervention or experienced a reduction in body weight to 20–25% of their pre-treatment value were considered to have reached a humane endpoint as defined by our ethical guidelines. Littermate-negative controls were used throughout all the experiments. Blinding was not implemented due to the necessity of genotyping to confirm GSK3 single or double knockout status. Importantly, animals of matched age and sex were selected for each experimental group, though the final datasets included specimens of both sexes and varying ages based on practical experimental requirements. This study used neonatal mice at postnatal days 7–8 (P7–P8) and adult mice at 8 weeks or older. Unless noted, all adult mice exceeded 25 g in weight. The experimental period was 7 to 10 days, according to the weight and status of the mice, and all procedures followed animal ethics regulations. If necessary, the mice were randomly assigned by computer-generated allocation in some of the mice experiments. All procedures were performed under the supervision of the animal center professionals.

### 2.2. Antibodies

The following antibodies were used for flow cytometry and immunofluorescence: anti-CD326 (EpCAM) (G8.8), anti-CD4 (GK1.5), anti-CD8 (53-6.7), anti-Fas (SA367H8), and anti-TCR-β (H57-597) were purchased from Biolegend (San Diego, CA, USA); anti-CD44 (IM7) and rat-anti mouse CD62L (MEL-14) were purchased from eBioscience (San Diego, CA, USA); anti-Ki67 (B56), anti-GL7 (Jo2), anti-CD45 R/B220 (RA3-6B2), and fixation buffer were purchased from BD Biosciences (San Jose, CA, USA); Goat-anti-rabbit AF488 (Cat# A-11034), Goat-anti-rabbit AF647 (Cat# A-21244), and Goat-anti-rabbit AF555 (Cat# A-21429) were purchased from Life Technologies (San Diego, CA, USA); and anti-Dclk1 (EPR6085) was purchased from Abcam (Cambridge, UK). Anti-Lyz1 and anti-ANNA1 were kind gifts from Professor Kairui Mao at Xiamen University. Anti-GSK3α (D80D1), anti-GSK3β (3D10), anti-GSK3α/β (D75D3), and anti-β-catenin (D10A8) were purchased from Cell Signaling Technology (Danvers, MA, USA).

### 2.3. Tissue Preparation and Histology

For tissue preparation, the mice were euthanized under anesthesia, and intestinal tissues were flushed with ice-cold PBS. The tissues were rolled into a “Swiss roll” and fixed by pre-fixation for 2 h, followed by overnight incubation in fixation buffer at 4 °C. After fixation, the tissues were rinsed with PBS for 10 min, cryoprotected in 30% sucrose for 24 h at 4 °C, embedded in Optimum Cutting Temperature (OCT) compound (Tissue-Tek, Tokyo, Japan), and frozen for sectioning.

For haematoxylin and eosin (H&E) staining, frozen sections were rinsed in ddH_2_O for 3 min, stained with hematoxylin for 20 s, and rinsed in running water for 3 min. The sections were then counterstained with eosin for 15 s, rinsed in water for 3 min, dehydrated through a graded ethanol series, cleared with Histo-Clear II (National Diagnostics, Atlanta, GA, USA) and mounted with Neutral Gum.

Periodic acid–Schiff (PAS) staining was performed on the frozen sections using a commercial kit (Solarbio, Beijing, China, Cat# G1281) according to the manufacturer’s instructions. Briefly, the sections were oxidized with periodic acid to generate aldehydes from carbohydrates, incubated with Schiff’s reagent to produce a magenta color in carbohydrate-rich structures, counterstained with haematoxylin to visualize nuclei, and then dehydrated, cleared, and mounted.

Alcian Blue staining was carried out using a staining kit (Sangon Biotech, Shanghai, China, Cat# E670106-0500) per the manufacturer’s protocol. In brief, frozen sections were stained with Alcian Blue solution to color acidic mucins blue, rinsed, counterstained with nuclear fast red to label nuclei, dehydrated through graded alcohols, cleared in xylene, and mounted for microscopy.

Immunofluorescence was performed as previously described [19], with minor modifications. The sections were washed with PBS for 10 min at room temperature (RT) and then permeabilized and blocked in 0.3% Triton X-100 and 1% normal mouse serum for 1 h at RT. The sections were incubated with primary antibodies in blocking solution overnight at 4 °C. After five 10 min PBS washes, fluorochrome-conjugated secondary antibodies were applied and incubated for 3 h at RT in the dark. Following another five 10 min PBS washes, the slides were mounted with Fluormount-G (SouthernBiotech, Birmingham, AL, USA) and coverslipped. Imaging was performed on a Leica TCS SP8 confocal microscope (Leica, Wetzlar, Germany) and analyzed using Imaris software (Bitplane, Version 10.2).

### 2.4. Quantitative RT-PCR

Total RNA was isolated from the distal 1 cm of small intestine using an RNA isolator (Vazyme, Nanjing, China). cDNA was synthesized from the extracted RNA with the Evo M-MLV RT Mix Kit (Accurate Biology, Changsha, China), followed by qPCR using Hieff^®^ qPCR SYBR Green Master Mix (No Rox; Yeasen, Beijing, China) on a Roche LightCycler 480 II system. Gene expression was quantified using the 2^−∆∆CT^ method, with β-actin as the reference gene. The primer sequences for mouse genes are listed in Supplementary Table S1.

### 2.5. Flow Cytometry

Single-cell suspensions were prepared from mouse lymph nodes following red blood cell lysis. The cells were stained with fluorochrome-conjugated antibodies against surface markers in phosphate-buffered saline (PBS) containing 0.5% fetal bovine serum (FBS) for 20 min at 4 °C in the dark. After staining, the cells were washed and resuspended in flow cytometry staining buffer for analysis.

For the flow cytometric analysis, the cells were initially gated on forward scatter area (FSC-A) versus side scatter area (SSC-A) to exclude debris. Single cells were then selected using side scatter height versus width (SSC-H/SSC-W) parameters, and live cells were identified by viability dye staining. Data acquisition was performed on a BD LSRFortessa™ X-20 flow cytometer (BD Biosciences, San Jose, CA, USA), and subsequent analysis was conducted using FlowJo software (version 10; Tree Star, BD Biosciences, San Jose, CA, USA).

### 2.6. Whole-Gut Transit Time

Intestinal motility was evaluated by measuring the whole-gut transit time. When mouse body weight decreased to approximately 85% of baseline, the animals received 100 μL of 6% carmine red dye suspended in 0.5% methylcellulose via oral gavage. Fecal pellets were subsequently collected and examined at 10 min intervals for the appearance of the red dye. The transit time was defined as the interval between gavage administration and the first excretion of carmine red-containing feces. The experiment was terminated if no dyed pellets were observed within 450 min, with this duration recorded as the transit time for such cases.

### 2.7. Nutritional Intervention with Liquid Diet

To examine whether nutritional support could improve outcomes under genetic deficiency, tamoxifen-induced intestinal epithelial Gsk3α/β knockout mice (iDKO) received a liquid diet intervention. Beginning immediately after tamoxifen induction, the mice were provided with a reconstituted liquid nutritional solution (Ensure enteral nutrition powder, 50 g/100 mL water) as their sole source of hydration and nutrition. The experimental group received additional caloric supplementation via daily oral gavage of the same liquid diet. Fresh solution was prepared daily, and mouse survival was monitored and recorded throughout the study period.

### 2.8. Western Blot

For the Western blot analysis, intestines were lysed in a buffer containing 20 mM Tris-HCl (pH 7.5), 150 mM NaCl, 1% Triton X-100, 1 mM EDTA, and 1 mM EGTA, supplemented with Halt^TM^ Protease and Phosphatase Inhibitor Cocktail (Thermo Fisher Scientific, Waltham, MA, USA). Proteins were resolved by 8% SDS-PAGE and transferred to a polyvinylidene fluoride (PVDF) membrane (Merck Millipore, Darmstadt, Germany). The membrane was incubated overnight at 4 °C with primary antibodies diluted in 1 × TBS (10 mM Tris-HCl, pH 8.0, 150 mM NaCl) containing 5% (*w*/*v*) BSA or non-fat milk. After three washes with TBST, the membrane was probed with HRP-conjugated goat anti-rabbit or anti-mouse secondary antibodies. Following three additional TBST washes, protein bands were detected using ECL Select Western Blotting Detection Reagent (GE Healthcare, Little Chalfont, UK) or Immobilon Western Chemiluminescent HRP Substrate (Merck Millipore, Darmstadt, Germany). according to the manufacturers’ instructions. Images were captured using an Amersham Imager 600 (GE Healthcare, Little Chalfont, UK).

### 2.9. Antibiotic Treatment (Abx)

To assess the role of gut microbiota in GSK3 deficiency-caused death, we depleted gut microbiota by oral gavage of an antibiotic cocktail (ampicillin, neomycin, metronidazole, and vancomycin; each 10 mg daily) for 5 consecutive days under mice survival, as previously described [20].

### 2.10. Image Acquisition

Bright-field images were acquired using an Olympus CX33 microscope (Olympus, Tokyo, Japan), and fluorescence images were acquired using a Leica TCS SP8 confocal microscope (Leica, Wetzlar, Germany). All bright-field images were captured with a 20× objective lens. Scale bars are indicated directly on the respective images.

### 2.11. Statistical Analysis

All the biological replicates (*n* = 3~11) in this study were included per group for analyses. Statistical analyses were performed using appropriate methods for each experimental design using GraphPad Prism software 8.0. Differences between two groups were assessed with two-tailed unpaired Student’s *t*-tests. For body weight measurement, data were analyzed by two-way ANOVA, followed by Sidak’s multiple comparisons test. Survival was compared using Kaplan–Meier curves and the Mantel–Cox log-rank test with 95% confidence intervals. All bar graphs show mean ± SEM. “*”, *p* < 0.05, “**”, *p* < 0.01, “***”, *p* < 0.001, “****”, *p* < 0.0001, ns indicates no significant differences. The data were organized and summarized using Adobe Illustrator software (Version 27.0).

## 3. Results

### 3.1. GSK3α or GSK3β Deletion Does Not Disrupt Intestinal Homeostasis and Tumorigenesis

**Figure 1:**
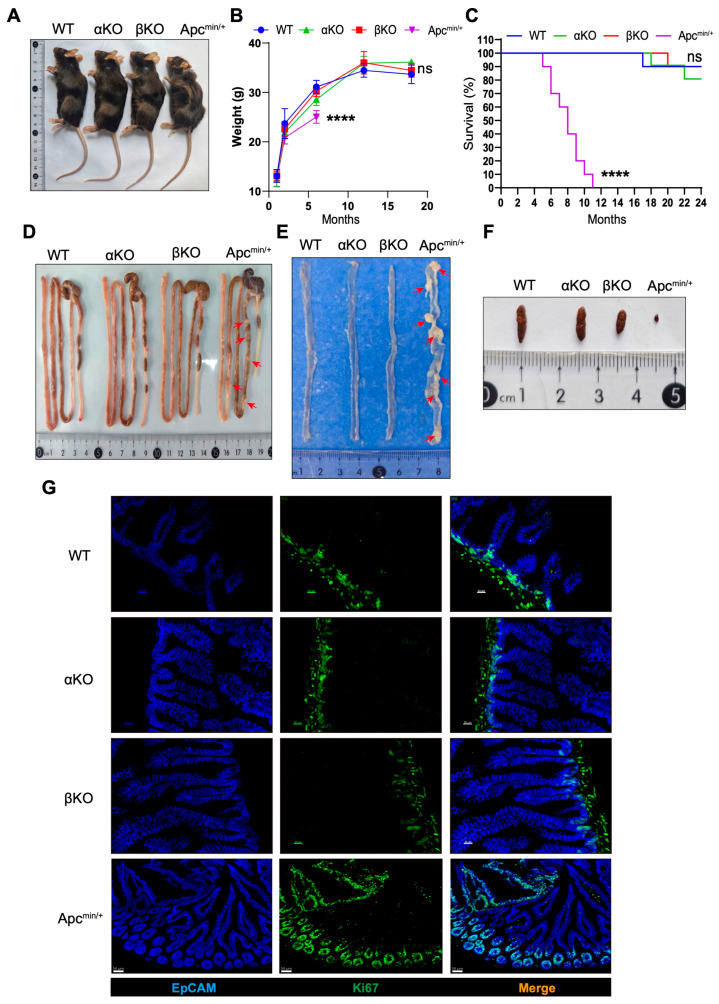
Deletion of GSK3α or GSK3β does not disturb intestinal homeostasis and tumorigenesis. WT, αKO, βKO, and Apc^Min/+^ mice were used for comparative analysis of body weight changes and fecal samples. Intestines were collected for morphological examination and immunofluorescence analysis. Each group contained at least 5 mice, and all data were collected from at least two independent experiments. (**A**) Representative images of WT, αKO, βKO, and Apc^Min/+^ mice. (**B**) Body weight of WT, αKO, βKO, and Apc^Min/+^ mice (*n* = 5). (**C**) Survival of WT, αKO, βKO, and Apc^min/+^ mice (*n* = 10). (**D**) Representative images of gut from WT, αKO, βKO, and Apc^Min/+^ mice. (**E**) Representative images of the small intestine from WT, αKO, βKO, and Apc^Min/+^ mice. (**F**) Representative images of mouse feces. (**G**) Representative immunofluorescence images of the small intestine from WT, αKO, βKO, and Apc^Min/+^ mice stained for EpCAM (blue) and Ki67 (green) (*n* = 6). Scale bar: 20 µm in WT, αKO, and βKO mice; 50 µm in Apc^Min/+^ mice. Arrow (red) shows hyperplastic polyps in Apc^min/+^ mice. Data are presented as mean ± SEM and statistical significance: **** *p* < 0.0001, ns, no significance.

GSK3 forms a regulatory complex with APC to modulate Wnt/β-catenin signaling, which is essential for intestinal homeostasis. To investigate the specific roles of GSK3α and GSK3β in intestinal epithelium, we generated intestinal-specific knockout mice (*Gsk3α*^fl/fl^; Villin-cre, termed αKO, and *Gsk3β*^fl/fl^; Villin-cre, termed βKO) and compared their phenotypes with the WT and Apc^Min/+^ controls. Both αKO and βKO mice displayed normal growth, body weight, fecal pellet formation, and survival. In contrast, Apc^Min/+^ mice developed progressive weight loss (nearly a 20% reduction), intestinal abnormalities (hyperplastic polyps), and 100% mortality within 4–12 months (Figure 1A,C). The histopathological examination revealed characteristic hyperplastic polyps in Apc^Min/+^ intestines, whereas αKO and βKO mice maintained normal intestinal architecture (Figure 1D–F). Epcam staining demonstrated villus elongation in αKO, βKO, and Apc^Min/+^ mice compared to the WT controls (Figure 1G), associated with enhanced β-catenin expression. Ki67 staining showed extensive proliferative clusters extending along the crypt–villus axis in Apc^Min/+^ intestines, while αKO and βKO mice exhibited normal proliferation restricted to crypt regions. Additional analyses confirmed the absence of polyp formation in both αKO and βKO mice based on a near two-year observation period. These results demonstrate that individual deficiency of GSK3α or GSK3β does not disrupt intestinal homeostasis or promote tumorigenesis.

### 3.2. Intestinal GSK3α and GSK3β Deficiency (DKO) Leads to Abnormal Intestinal Development and Perinatal Death

**Figure 2:**
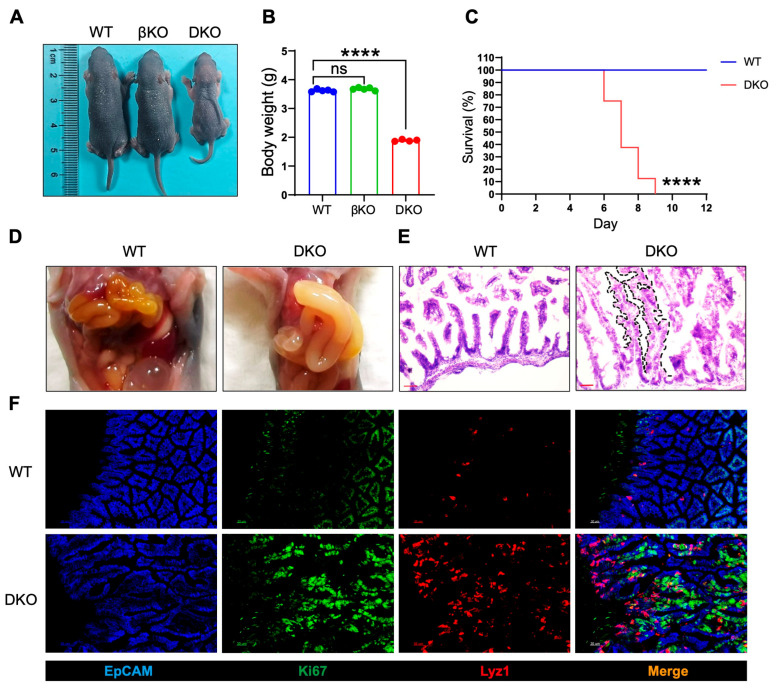
GSK3 deficiency (DKO) leads to abnormal intestinal development and perinatal death. WT, βKO, and DKO mice were used for comparative analysis of body weight changes and survival. Intestines were collected for morphological examination and immunofluorescence analysis. Each group contained at least 5 mice, and all data were collected from at least two independent experiments. (**A**) Images of postnatal day 6 (P6) WT, βKO, and DKO mice. (**B**) Body weight of postnatal day 6 (P6) WT, βKO, and DKO mice (WT and βKO mice, *n* = 5; DKO mice, *n* = 4). (**C**) Survival of WT and DKO mice (*n* = 8). (**D**) Representative images of gut from WT and DKO mice. (**E**) H&E-stained sections of the small intestine from WT and DKO mice (*n* = 6). Scale bar, 100 µm. The black dashed lines delineate the elongated villi and crypt-like structures. (**F**) Representative immunofluorescence images of the small intestine from WT and DKO mice stained for EpCAM (blue), Ki67 (green), and Lyz1 (red) (*n* = 6). Scale bar, 30 µm. Data are presented as mean ± SEM and statistical significance: **** *p* < 0.0001, ns, no significance.

Although intestinal deletion of GSK3α and GSK3β did not cause overt abnormalities, we hypothesized that functional compensation between the two isoforms might occur. We, therefore, generated double-knockout (*Gsk*3α^fl/fl^; *Gsk3*β^fl/fl^; Villin-cre, DKO) mice and confirmed by Western blot assay (Figure S1). The number of DKO offspring deviated from Mendelian expectations, indicating embryonic or perinatal lethality. DKO pups exhibited morphological changes (weakness) and reduced body weight (nearly a 50% decrease) by postnatal day 5, with 100% mortality occurring between days 7 and 9 (Figure 2A–C). At day 7, the DKO intestine displayed a significantly increased intestinal diameter (nearly a two-fold increase) and severe bowel swelling, consistent with a megacolon pathology (Figure 2D). These results underscore the essential role of GSK3 in maintaining intestinal homeostasis. Further analysis revealed that DKO mice had markedly elongated villi with branching and crypt-like structures, suggesting disrupted localization and function of intestinal stem cells (Figure 2E). Under normal conditions, intestinal stem cells receive WNT signals from Paneth cells and give rise to transient amplifying cells, which differentiate into various intestinal cell types. Examination of Lyz1+ Paneth cells and Ki67+ proliferating cells showed a significant increase in GSK3-deficient intestines. Notably, Lyz1+ Paneth cells were not confined to the crypt base but had migrated toward the villus tips, while transient amplifying cells were broadly distributed along the villi (Figure 2F). These findings indicate that GSK3 deficiency in the intestine resulted in lethality, disrupted homeostasis of intestinal stem and Paneth cells, and structural abnormalities in crypts and villi.

### 3.3. Inducible Deletion of GSK3 in Adult Mice Results in Intestinal Abnormalities and Lethality

**Figure 3:**
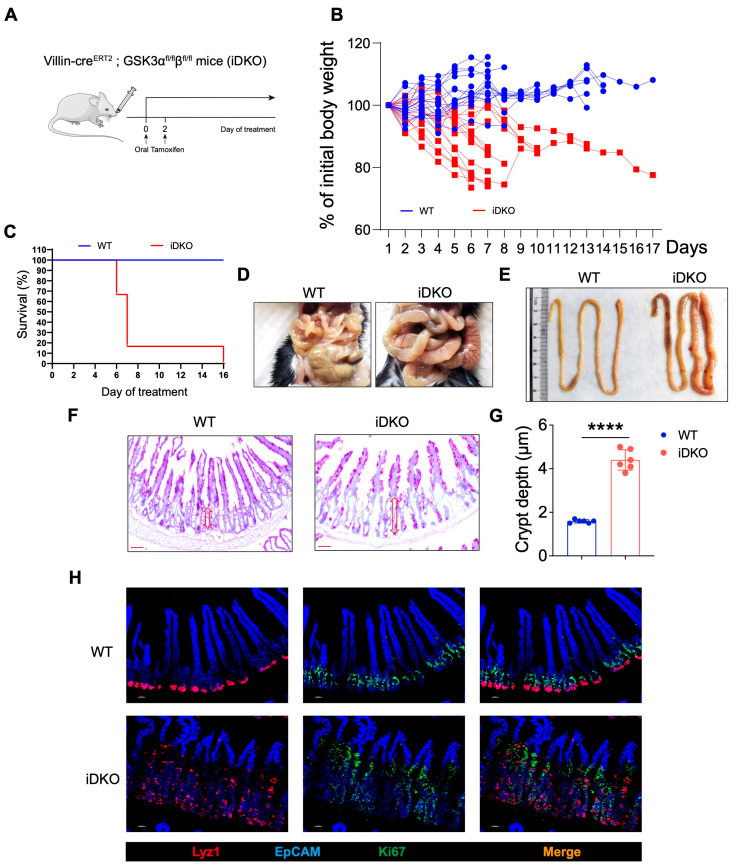
Inducible GSK3 deficiency causes intestinal abnormalities and lethality. WT and iDKO mice were used for comparative analysis of body weight changes and survival. Intestines were collected for morphological examination and immunofluorescence analysis. Each group contained at least 5 mice, and all data were collected from at least two independent experiments. (**A**) Experimental scheme. (**B**) Body weight of WT and iDKO (*n* = 15). (**C**) Survival of WT and iDKO mice (*n* = 6). (**D**,**E**) Representative image of gut from WT and iDKO mice. (**F**) PAS-stained sections of the small intestine from WT and DKO mice (*n* = 6). Scale bar, 50 µm. Arrow (red) shows the length of crypt area. (**G**) Crypt depth in the small intestine of WT and iDKO mice (*n* = 6). (**H**) Representative immunofluorescence images of the small intestine from WT and iDKO mice stained for EpCAM (blue), Ki67 (green), and Lyz1 (red) (*n* = 6). Scale bar, 30 µm. Data are presented as mean ± SEM and statistical significance. **** *p* < 0.0001.

Perinatal mortality in DKO mice limited subsequent intestinal development, which prevented further analysis of feeding and intestinal function. To address this, we crossed Villin-ERT2cre mice with *Gsk3*-floxed mice to generate inducible double-knockout (iDKO) mice. Tamoxifen was administered orally to adult mice to induce Gsk3 deletion (Figure 3A). The results showed that iDKO mice showed rapid weight loss (nearly a 20% reduction) and died within 7–10 days following Gsk3 deletion (Figure 3B,C), indicating that GSK3 deficiency still induces lethality in adult mice, even after normal intestinal development, and highlighting the essential role of Gsk3 in the intestine. Furthermore, the intestinal morphology of Gsk3 deficient mice exhibited a megacolon-like phenotype (Figure 3D,E), consistent with previous observations in DKO mice. Although villus length did not increase significantly in adult iDKO mice, the crypt area was still enlarged, and villi displayed branching and crypt-like structures, suggesting disrupted localization and function of intestinal stem cells following Gsk3 deletion (Figure 3F). The analysis of Lyz1+ Paneth cells and Ki67+ transient amplifying cells showed a significant increase in both cell types in iDKO intestines. Lyz1+ Paneth cells were no longer confined to the crypt base but had migrated toward the villus tips, while transient amplifying cells were distributed along the villi (Figure 3G). Intestinal abnormalities can directly or indirectly cause functional impairments, leading to digestive disorders, compromised barrier function, dehydration, infection, or severe diarrhea. Collectively, these results demonstrate that GSK3 is essential for maintaining normal intestinal function in adult mice.

### 3.4. Intestinal GSK3 Deficiency Dampens Multi-Organs and Slightly Affects Feeding Capacity

**Figure 4:**
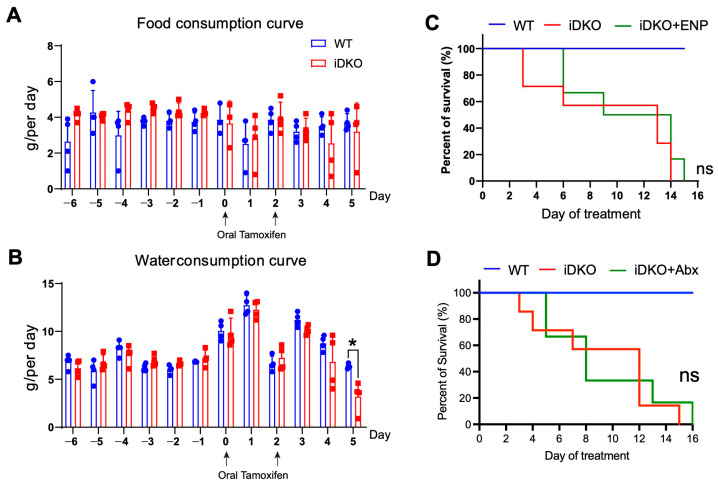
GSK3 deficiency slightly affects feeding capacity. WT and iDKO mice were used for comparative analysis of food and water consumption, and iDKO mice received ENP and antibiotic treatment. Each group contained at least 4 mice, and all data were collected from at least two independent experiments. (**A**,**B**) Food (**A**) and water (**B**) intake in WT and iDKO mice (*n* = 4). (**C**) Survival of WT, iDKO, and iDKO + ENP mice (WT and iDKO mice, *n* = 7; iDKO + ENP mice, *n* = 6). ENP: enteral nutritional powder. (**D**) Survival of WT, iDKO, and iDKO + Abx mice (WT and iDKO mice, *n* = 7; iDKO + Abx mice, *n* = 6). Abx: antibiotic treatment. Data are presented as mean ± SEM and statistical significance: *, *p* < 0.05; ns, no significance.

To investigate the cause of mortality after GSK3 deletion, we first examined various organs in iDKO mice. Despite intestinal megacolon, the liver, spleen, and kidney showed significant reductions in both size and weight (Figure S2A,B). As the liver and kidneys are key metabolic organs and the spleen plays a crucial role in maintaining immune homeostasis, these findings indicate that intestinal GSK3 deficiency leads to multi-organ abnormalities, potentially through mechanisms such as impaired food intake, dehydration, water–electrolyte imbalances, or systemic hypoperfusion. We initially hypothesized that reduced dietary intake due to GSK3 deficiency might cause malnutrition. However, measurement of feeding capacity revealed that GSK3 deletion did not affect the ability of mice to consume food and water, except for a decrease in water consumption on day 5. These results could not explain the observed weight loss and mortality following GSK3 deletion (Figure 4A,B). We next considered whether the weakened condition of iDKO mice impaired their ability to swallow effectively, leading to weight loss. To test this, we administered enteral nutrients (enteral nutritional powder, termed ENP) to iDKO mice via gavage in an attempt to rescue them. However, this nutritional support did not significantly prolong survival (Figure 4C). Furthermore, we observed a striking difference in fecal color between WT and iDKO mice, suggesting that GSK3 deficiency may also alter gut microbiota (Figure S2C). Since microbial disruption can impair normal intestinal function, we administered a 5-day antibiotic treatment to iDKO mice. However, this treatment still failed to prevent their mortality (Figure 4D). These results suggest that both food and water intake and gut microbiota are not the primary cause of mortality in GSK3-deficient mice, indicating that other critical factors are involved in the death of iDKO mice.

### 3.5. GSK3 Deficiency Affects the Intestinal Cell Niche and Absorptive and Peristaltic Function

**Figure 5:**
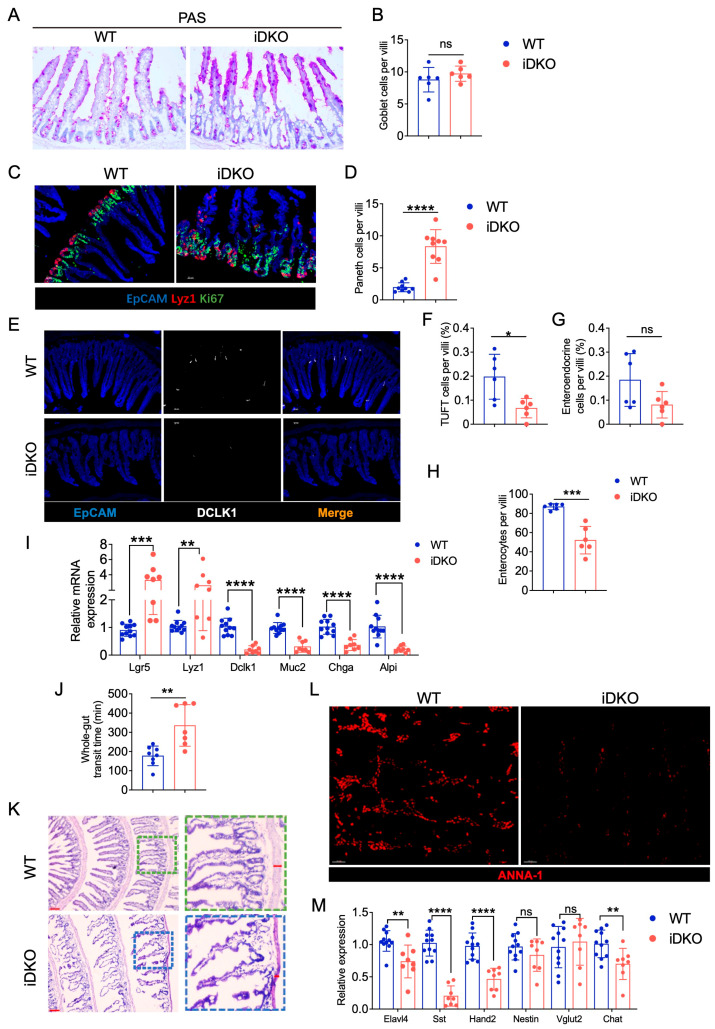
Deletion of GSK3 disturbs intestinal cell niche and absorptive and peristaltic function. WT and iDKO mice were used for comparative analysis of intestinal cell niche and function. Intestines were collected for morphological examination, immunofluorescence, and mRNA analysis. Each group contained at least 5 mice, and all data were collected from at least two independent experiments. (**A**,**B**) Goblet cells from PAS-stained sections of the small intestine from WT and iDKO mice (*n* = 6) (**A**) and analysis of the total number of goblet cells per villi (**B**); Scale bar, 50 µm. (**C**,**D**) Representative immunofluorescence images of the small intestine from WT and iDKO mice stained for EpCAM (blue), Ki67 (green), and Lyz1 (red) (*n* = 9) (**C**) and analysis of the total number (**D**) of Paneth cells per villi. Scale bar, 30 µm. (**E**–**G**) Representative immunofluorescence images (**E**) of the small intestine from WT and iDKO mice stained for DLCK1 (white), ChaA (red), and EpCAM (blue) (*n* = 6) and analysis of the frequency of tuft cells (**F**) and enteroendocrine cells (**G**) per villi (*n* = 6); Scale bar, 30 µm. (**H**) the numbers of enterocytes are quantified as all villus cells excluding goblet cells, Paneth cells, tuft cells, and enteroendocrine cells (*n* = 6). (**I**) RT-qPCR to detect the mRNA expression of *Lgr5*, *Lyz1*, *Dlck1*, *Muc2*, *Chga*, and *Alpi* in intestine from WT and iDKO mice (*n* = 8~11). (**J**) Analysis of the whole-gut transit time from WT and iDKO mice after tamoxifen administration (*n* = 6–8). (**K**) H&E-stained sections of the small intestine from WT and DKO mice (*n* = 6); Scale bar, 200 µm (left) and 30 µm (right). (**L**) Representative immunofluorescence images of the muscular layer in small intestine from WT and iDKO mice stained for ANNA-1 (red); Scale bar, 30 µm. (**M**) RT-qPCR to detect the mRNA expression of *Elavl4*, *Sst*, *Hand2*, *Nestin*, *Vglut2*, and *Chat* in muscular layer of small intestine from WT and iDKO mice (*n* = 8~11). Data are presented as mean ± SEM and statistical significance: *, *p* < 0.05; **, *p* < 0.01; ***, *p* < 0.001; **** *p* < 0.0001. ns, no significance.

To investigate whether Gsk3 deletion in intestinal epithelial cells affects digestive and absorptive functions, we analyzed the distribution and abundance of various functional cell types. We examined goblet cells, tuft cells, enteroendocrine cells, and enterocytes, all of which are crucial for mucus secretion, hormone release, and nutrient absorption. Compared with WT mice, iDKO mice showed no significant change in goblet cell numbers (Figure 5A,B). However, while goblet cells were evenly distributed along the villi in WT mice, they became concentrated at the villus tips in iDKO mice. Furthermore, in addition to the previously observed Paneth cell alterations (Figure 5C,D), the numbers of DCLK1⁺ (doublecortin-like kinase 1) tuft cells and ChgA⁺ (chromogranin A) enteroendocrine cells were significantly reduced (Figure 5E–G). By quantifying all villus cells and excluding those above cell types, we determined that the number of enterocytes was significantly reduced (Figure 5H), indicating impaired nutrient absorption. At last, mRNA analysis of intestinal epithelial cell markers revealed that iDKO mice exhibited increased *Lgr5*^+^ stem cells and Paneth cells but decreased levels of other cell types, including goblet cells marked by *Muc2* (Figure 5I). These results demonstrate that GSK3 deficiency disrupts the niche of functional intestinal cells and compromises absorptive capacity.

Peristalsis is another critical factor in intestinal digestion and absorption, regulated by the muscular layers and enteric neurons. Using carmine red gavage to track gastrointestinal transit, we observed significantly delayed excretion in iDKO mice compared with the WT controls (Figure 5J), indicating defective intestinal motility. Histological analysis revealed a markedly thinner muscular layer in iDKO mice (Figure 5K). Since intestinal motility is neurally regulated, we used ANNA-1 to label neuronal nuclei and found that Gsk3 deletion significantly reduced ANNA-1 expression in the muscular layer (Figure 5L). RT-qPCR further showed decreased mRNA expression of the pan-neuronal marker *Elavl4* as well as significant reductions in *Sst* and *Hand2* in neurons (Figure 5M), which are known to regulate intestinal motility. Together, these results indicate that intestine-specific deletion of Gsk3 disturbs the intestinal niche, leads to a significant loss of enterocytes, impairs intestinal motility, and reduces populations of key neurons regulating motor and absorptive functions, collectively compromising intestinal absorption.

### 3.6. GSK3 Deficiency Does Not Affect Immune Homeostasis in Mesenteric Lymph Nodes

**Figure 6:**
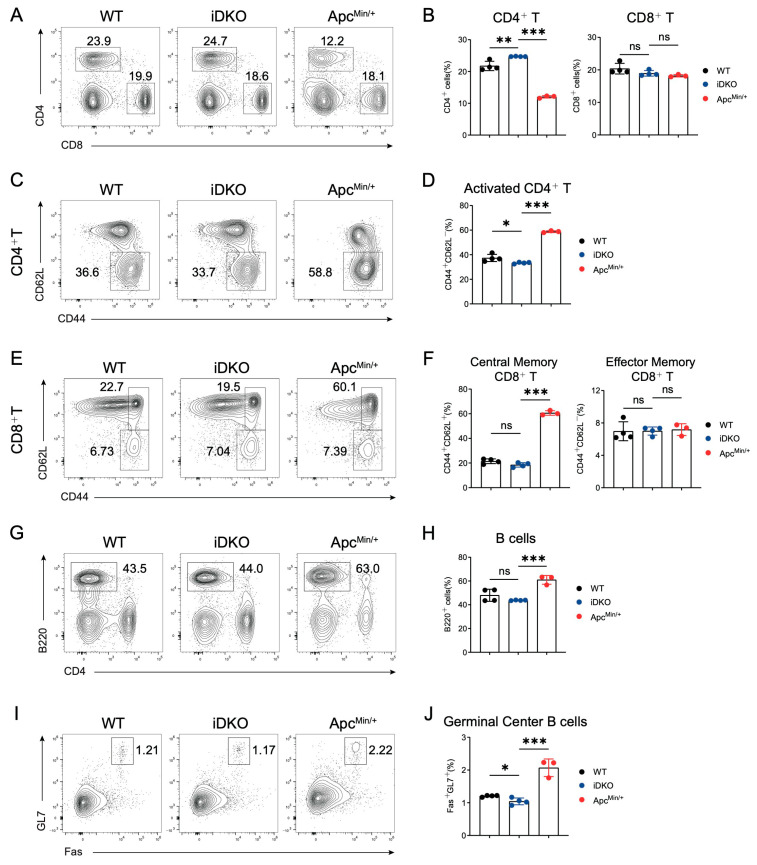
GSK3 deficiency does not affect intestinal immune homeostasis in mLNs. WT, iDKO, and Apc^min/+^ mice were used for immune cell analysis in mLNs. Each group contained at least 3 mice. (**A**,**B**) CD4+ T and CD8+ T cells in mLNs from WT, iDKO, and Apc^Min/+^ mice after tamoxifen administration (**A**) and analysis of the percentage of CD4+ T and CD8+T cells (**B**). (**C**,**D**) CD4+ T cell activation (CD44+CD62L-) in mLNs from WT, iDKO, and Apc^Min/+^ mice (**C**) and analysis of the percentage of T cell activation (**D**). (**E**,**F**) CD8+ T cell activation (central memory T cells, CD44+CD62L+; effector memory T cells, CD44+CD62L-) in mLNs from WT, iDKO, and Apc^Min/+^ mice (**C**) and analysis of the percentage of T cell activation (**F**). (**G**,**H**) B cells (B220+) in mLNs from WT, iDKO, and Apc^Min/+^ mice (**G**) and analysis of the percentage of B cells (**H**). (**I**,**J**) Germinal center B cells (Fas+GL7+, GC B) in mLNs from WT, iDKO, and Apc^Min/+^ mice (**C**) and analysis of the percentage of GC B cells (**J**). Data are presented as mean ± SEM and statistical significance: *, *p* < 0.05; **, *p* < 0.01; ***, *p* < 0.001, ns, no significance.

Within the intestine, the mucosal barrier, immune cells, and microbiota coexist for balance. Whether the altered intestinal cell niche and functional impairments caused by GSK3 deletion could trigger immune dysregulation and disrupt immune homeostasis remains unclear. We collected mesenteric lymph nodes (mLNs) from WT, DKO, and Apc^Min/+^ mice and analyzed T cell proportions and activation as well as germinal center responses in B cells. Unlike Apc^Min/+^ mice, which exhibited reduced CD4+ T cell proportions, both WT and DKO mice showed no changes in the ratios of CD4+ T, CD8+ T. In addition, T cell activation levels were also comparable in mLNs (Figure 6A–F). In contrast to the elevated B cell proportions and enhanced germinal center responses (Fas+GL7+) observed in Apc^Min/+^ mice, neither B cell frequency nor germinal center reaction were normal in mLNs between WT and iDKO mice (Figure 6G–J). Based on these findings, we conclude that the disrupted niche and function of intestinal *Gsk3* deficiency are in a cell-intrinsic manner rather than external immune alterations in mLNs. In comparison, intestinal polyp formation in Apc^Min/+^ mice may result from a combination of intrinsic cellular defects and extrinsic immune abnormalities.

### 3.7. GSK3 Deficiency Promotes an Increase and Redistribution of β-Catenin-Positive Cells

**Figure 7:**
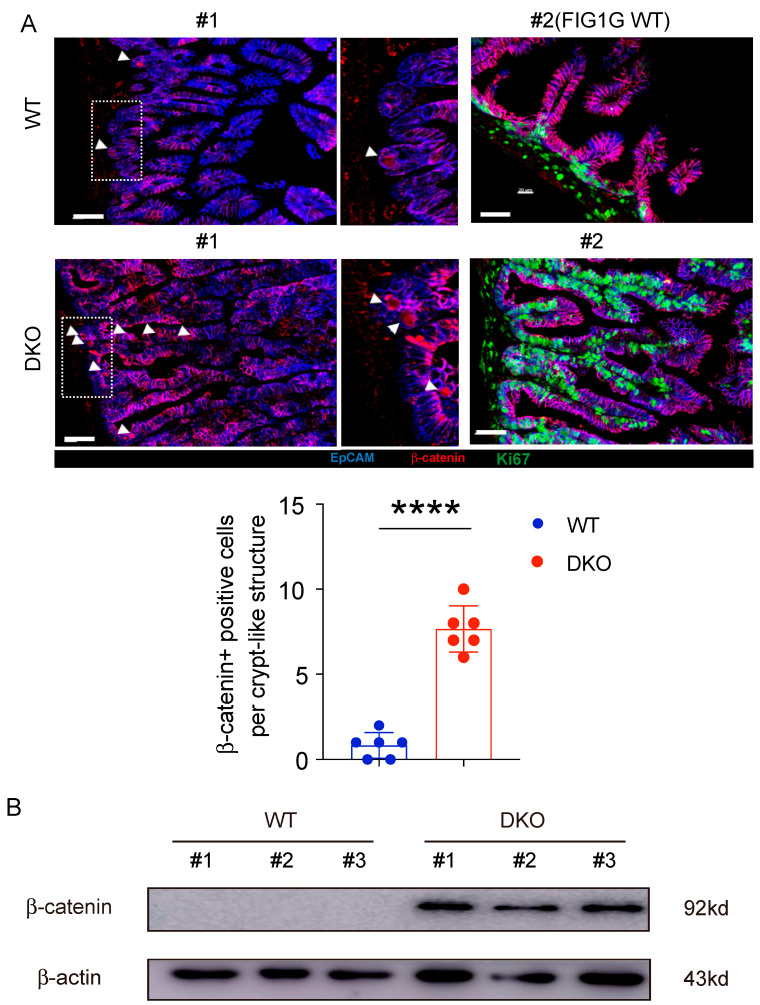
Deletion of GSK3 in intestine enhances β-catenin-positive cells. WT and DKO mice were used for comparative analysis of β-catenin-positive cells. Intestines were collected for immunofluorescence and Western blot analysis. Each group contained at least 3 mice, and all data were collected from at least two independent experiments. (**A**) Representative immunofluorescence images (up) and β-catenin-positive cells (down) of the small intestine from WT and iDKO mice stained for Ki67 (green), β-catenin (red), and EpCAM (blue) (*n* = 6). Scale bar, 30 µm. #1 and #2 represent 2 WT or iDKO mice. Notably, #2 WT was the same view related to Figure 1 G WT controls. Arrow (white) shows β-catenin expression in nuclei. (**B**) β-catenin expression was detected in intestine from WT and DKO mice using Western blot analysis. #1, #2, and #3 represent 3 WT or iDKO mice. Data are presented as mean ± SEM and statistical significance: ****, p < 0.0001.

As a key negative regulator of the WNT/β-catenin signaling pathway, we hypothesize that GSK3 precisely regulates β-catenin expression to enable rapid repairment of intestinal epithelial cells during apoptosis or injury, thereby maintaining the structural integrity and normal function of the intestinal epithelium. As shown in Figure 7A, we observed significantly more β-catenin nuclear-positive cells in the intestinal epithelium of DKO mice compared with the WT controls. In contrast to WT intestines, where positive cells were mainly confined to the crypt base, β-catenin-positive cells in DKO intestines were present not only in the crypts but also scattered along the intestinal villi. This distribution contributed to the formation of crypt-like structures on the villi of iDKO mice. Furthermore, we found a significant increase in β-catenin expression in DKO intestine by protein analysis (Figure 7B). These results demonstrate that GSK3 deletion significantly enhances the β-catenin expression, promoting the migration of stem cells and Paneth cells from the crypt base toward the villus tips and resulting in the formation of crypt-like structures, which disturbs the niche of the intestine.

### 3.8. β-Catenin Deletion Ameliorates Intestinal Cell Proliferation and Restores the Intestinal Niche by GSK3 Deletion

**Figure 8:**
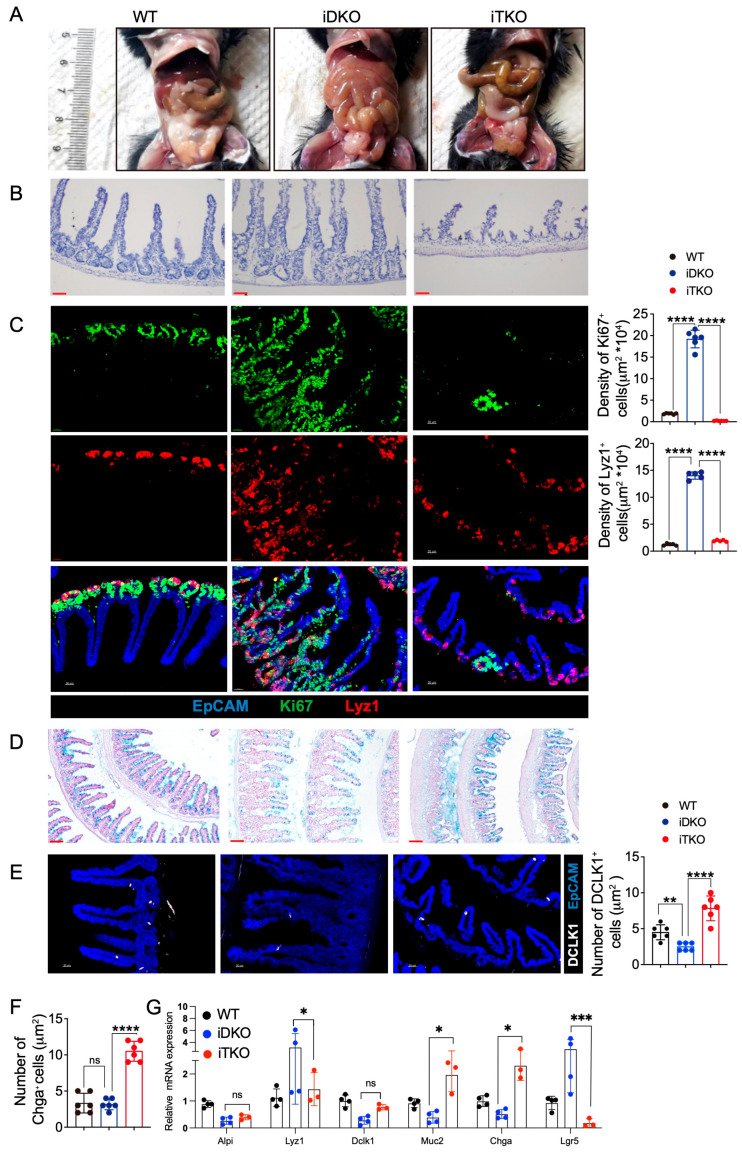
β-catenin deletion ameliorates intestinal cell proliferation and restores the intestinal niche by GSK3 deletion. WT, iDKO, and iTKO mice were used for comparative analysis of intestinal cell niche and function. Intestines were collected for morphological examination, immunofluorescence, and mRNA analysis. Each group contained at least 3 mice, and all data were collected from at least two independent experiments. (**A**) Representative image of WT, iDKO, and iTKO mice. (**B**) H&E-stained sections of the small intestine from WT, iDKO, and iTKO mice (*n* = 6). Scale bar, 30 µm. (**C**) Representative immunofluorescence images and cell numbers of the small intestine from WT, iDKO, and iTKO mice stained for EpCAM (blue), Ki67 (green), and Lyz1 (red) (*n* = 6). Scale bar, 30 µm. (**D**) Detection of goblet cells by Alcian Blue Staining in the sections of the small intestine from WT, iDKO, and iTKO mice; Scale bar, 100 µm. (**E**) Representative immunofluorescence images and cell numbers of the small intestine from WT, iDKO, and iTKO mice stained for DLCK1 (white) and EpCAM (blue) (*n* = 6); Scale bar, 30 µm. (**F**) Cell numbers of enteroendocrine cells in WT, iDKO, and iTKO mice (Chga+). (**G**) RT-qPCR to detect the mRNA expression of Lgr5, Lyz1, Dlck1, Muc2, Chga, and Alpi in intestine from WT, iDKO, and iTKO mice (*n* = 3~4). Data are presented as mean ± SEM and statistical significance: *, *p* < 0.05; **, *p* < 0.01; ***, *p* < 0.001; ****, *p* < 0.0001; ns, no significance.

To determine whether increased β-catenin directly causes the abnormal intestinal cell niche and impaired absorptive and motility functions resulting from GSK3 deficiency, we generated triple-knockout (*Gsk3α*^fl/fl^; *Gsk3β*^fl/fl^; *Ctnnb1*^fl/fl^; Villin-ERT2cre, iTKO) mice. The deletion efficiency of β-catenin was confirmed by the Western blot assay (Figure S3). Unlike the intestinal swelling and megacolon observed in iDKO mice, iTKO intestines did not show swelling (Figure 8A). H&E staining revealed that the elongated villi in iDKO mice were significantly shorter in iTKO mice, and the muscular layer of iTKO intestines was restored to a thicker state, indicating the recovery of peristalsis (Figure 8B).

Further analysis showed markedly reduced numbers of Paneth cells and transient amplifying cells in iTKO intestines. Additionally, Lyz1⁺ Paneth cells were largely confined to the crypt base, with only a few migrating toward the villus tips (Figure 8C). These findings indicate that β-catenin deletion significantly suppressed cell proliferation and partially restored Paneth cell localization. We next analyzed the number and distribution of tuft cells, enteroendocrine cells, and goblet cells. As shown in Figure 8D, compared to iDKO mice, iTKO mice had significantly more goblet cells, which were redistributed uniformly along the villi. iTKO mice also exhibited increased numbers of enteroendocrine cells and tuft cells (Figure 8E,F). RT-qPCR analysis of intestinal epithelial cell markers confirmed that iTKO mice had fewer Paneth cells and Lgr5⁺ stem cells compared to iDKO mice, while goblet cells and enteroendocrine cells were increased (Figure 8G). These data demonstrate that deletion of β-catenin expression partially restores the number and distribution of functional intestinal cells as well as goblet cell secretion, which improves the intestinal niche. However, due to the significant reduction in intestinal stem cells, the mice ultimately still died.

## 4. Discussion

Our findings demonstrate that GSK3 is essential for maintaining the intestinal cell niche and regulating digestive, absorptive, and peristaltic functions. Under physiological conditions, Paneth cell-derived Wnt ligands activate AKT activation, which inhibits the ability of GSK3 and results in β-catenin accumulation, promoting the transcription factors TCF1/LEF1 to drive intestinal stem cell proliferation and differentiation into functional cell types, which supports regular self-renewal and intestinal function [21,22]. However, excessive β-catenin activation enhances intestine cell hyperproliferation and tumorigenesis. To maintain intestine homeostasis, active GSK3 subsequently downregulates β-catenin, ensuring controlled self-renewal every 5–7 days. Except for the role of GSK3/β-catenin in controlling tumors, in intestinal GSK3-deficient mice, sustained β-catenin overexpression resulted in crypt-like structures, expanded Paneth cells, and loss of functional intestinal cells, disrupting tissue architecture and niche organization. These defects impaired intestinal absorption, digestion, and motility. GSK3 deficiency leads to the activation of β-catenin signaling, which promotes the expression of downstream targets including TCF1, LEF1, c-MYC, and cyclin D1. These factors are critical for Paneth cell proliferation and survival [23]. However, excessive β-catenin signaling drives hyperproliferation while compromising normal differentiation into functional intestinal cells. This imbalance limits digestive and absorptive functions, ultimately promoting intestinal tumorigenesis or defective function, as observed in Apc^min/+^ mice [24]. Although β-catenin deletion did not rescue viability due to impaired stem cell development, it partially normalized Paneth cell numbers and restored goblet cell distribution and tuft cell populations, which repaired niche organization. Thus, our work elucidates the dual regulatory role of GSK3 in intestinal stem cell dynamics, niche maintenance, and functional cell differentiation.

Complete loss of GSK3 causes intestinal defects, whereas individual deletion of GSK3α or GSK3β preserves normal intestinal structure and survival without tumor development. This indicates functional redundancy between the two isoforms. Both GSK3α and GSK3β share a highly conserved kinase domain, which nears 98% sequence homology, but diverge considerably in their non-catalytic N- and C-terminal regions [13]. Although both have similar roles in regulating metabolism, proliferation, differentiation, and apoptosis, their functions are not always interchangeable. Our previous work revealed that deletion of GSK3α or GSK3β does not affect thymic mature T cell egress, whereas double knockout (DKO) inhibits mature thymocyte egress and induces thymic apoptosis, indicating a context-dependent compensatory mechanism [18]. In CD8⁺ T cells, inhibition of GSK3β enhances cytotoxicity, but total GSK3 deficiency promotes T cell exhaustion and accelerates tumor growth, underscoring the functional divergence between single and double deletion [25,26]. Most studies focus on GSK3β but mislead GSK3α, resulting in limited conclusions to investigate the role of GSK3 in physiological process. However, there is evidence that indicates clear functional differences between the two isoforms. For example, in acute myeloid leukemia (AML), GSK3α but not GSK3β suppresses c-myc and cyclin D1 to maintain stemness [27]. In cardiac pathology, GSK3α enhances cardiomyocyte proliferation and ameliorates heart failure, while GSK3β is associated with cardiac hypertrophy and fibrosis [28,29,30]. Thus, future research on GSK3 should account for both the common and unique functions of GSK3α and GSK3β.

Recent studies have reported that GSK3 participates in intestinal homeostasis by regulating the epithelial barrier, host–microbiota interactions, and tight junction protein expression. In terms of epithelial barrier function, inhibition of GSK3 via small-molecule inhibitors or siRNA suppresses the transcription and expression of tight junction proteins, such as occludin, claudin-1, and E-cadherin, thereby impairing paracellular permeability and increasing intestinal leakage [17]. Under inflammatory conditions, such as IBD (inflammatory bowel disease), activated GSK3β enhances the NF-κB (nuclear factor kappa B) and MAPK (mitogen-activated protein kinase) signaling pathways, promoting the secretion of pro-inflammatory cytokines like IL-6 (interleukin-6) and TNF-α (tumor necrosis factor-alpha) and exacerbating intestinal inflammation [31]. Similarly, trimethylamine N-oxide (TMAO), a metabolite of dietary choline by gut microbes, activates GSK3β by reducing its phosphorylation at Ser9. Activated GSK3β then phosphorylates PSD95, leading to synaptic dysfunction [32]. Furthermore, β-catenin also participates in intercellular adhesion by binding to E-cadherin. Abnormal accumulation of β-catenin disrupts adheren junctions, promoting tumor cell infiltration and metastasis [33]. Together with our findings, GSK3 regulates intestinal stem cell development and niche maintenance. These results underscore the critical role of GSK3 and its downstream pathways in intestinal physiology and pathology.

Although both GSK3 deletion and the Apc^Min/+^ mutation result in β-catenin upregulation, they produce markedly different phenotypes. GSK3 deficiency leads to lethality, associated with disruption of the intestinal niche and loss of absorptive function. In contrast, Apc^Min/+^ mice exhibit normal survival and body weight in the early stage but develop intestinal polyps later in life. These findings indicate that the precise regulation of β-catenin expression levels is essential for intestinal development, homeostasis, function, and carcinogenesis. The following are related to β-catenin expression: (1) absence of β-catenin prevents intestinal stem cell development and disrupts the organized arrangement of villus epithelial cells [34]; (2) when β-catenin expression is modulated by GSK3 or APC, its finely tuned regulation guides intestinal stem cell development and differentiation into diverse functional cell types, thereby sustaining a proper niche and normal intestinal function [8]; (3) Apc^Min/+^ mice carry a heterozygous Apc gene mutation that produces a truncated APC protein, which impairs the ability of APC to bind β-catenin and facilitate its degradation, leading to β-catenin accumulation and activation of Wnt target genes such as c-myc, cyclin D1, and matrilysin [35,36], and consequently, intestinal epithelial proliferation is enhanced, resulting in multiple adenomatous polyps; (4) complete GSK3 loss causes substantial β-catenin accumulation, promoting Paneth cell proliferation and mislocalization. This disrupts the intestinal niche and absorptive capacity, ultimately causing lethality. Similarly, Apc^Min/Min^ mice show embryonic lethality due to excessive β-catenin accumulation that blocks embryonic stem cell differentiation, leading to embryonal carcinoma and embryonic death [37,38]. Notably, although individual deletion of GSK3α or GSK3β upregulates β-catenin, neither manipulation induces tumor or polyp formation. This suggests that GSK3 may regulate intestinal function through β-catenin-independent mechanisms, such as NF-κB and mTOR (mammalian target of rapamycin) signals, of which NF-κB has been reported to determine Paneth versus goblet cell fate decision in the small intestine [39], or mTOR-related autophagy controls the intestinal epithelial barrier [40].

The survival of enteric neurons depends on a complex tissue microenvironment maintained by diverse cells, signaling molecules, and structural components. GSK3 deletion impairs the intestinal cell niche and epithelial cell function, which may disrupt enteric neurons via two potential mechanisms. Firstly, it could reduce the secretion of epithelium-derived neurotrophic factors and compromise the intestinal barrier, allowing harmful substances to infiltrate the tissue and perturb the neuronal microenvironment, ultimately triggering enteric neuronal apoptosis [41]. Secondly, given that intestinal epithelial cells communicate directly with enteric neurons, for instance, through neurotransmitter release, GSK3 deficiency may also disrupt this epithelial–neuronal crosstalk, leading to neuronal dysfunction [42].

Our study has several limitations. Although we have clarified the role of GSK3 in intestinal homeostasis, the key underlying mechanisms remain elusive. For example, how GSK3 regulates Paneth cell survival and localization as well as its impact on the differentiation and function of various intestinal epithelial cell types are still poorly understood. Moreover, while GSK3 is known to regulate neuronal activity, its potential influence on intestinal function via appetite regulation or enteric neural circuits warrants further exploration. Finally, in developing GSK3 inhibitors for therapeutic applications, it will be crucial to evaluate drug effects on intestinal absorption, digestion, and motility post-treatment. These aspects represent important avenues for future investigation.

## 5. Conclusions

Our study reveals the critical role of GSK3, a key negative regulator of the WNT/β-catenin signaling pathway, in maintaining the intestinal cellular niche and regulating absorption and motility. Deletion of GSK3 leads to lethality in mice, characterized by increased numbers and mislocalization of intestinal stem cells and Paneth cells, disruption of the functional cell niche, and severe impairment of digestive functions. GSK3 deficiency results in marked accumulation of β-catenin in the intestine, while subsequent deletion of β-catenin partially reverses the expansion and aberrant localization of stem and Paneth cells by GSK3 deletion and increases the proportions of goblet cells, tuft cells, and enteroendocrine cells, thereby restoring the intestinal cellular niche. These findings identify GSK3 as a potential therapeutic target for intestinal disorders and digestive dysfunction.

## Data Availability

All data of this study are available on reasonable request from the corresponding author Chenfeng Liu.

## Supplementary Materials

The following supporting information can be downloaded at: https://www.mdpi.com/article/10.3390/biology14111551/s1, Table S1: Primers for RT-qPCR; Figure S1: Deletion efficiency of GSK3α/β in DKO mice. Figure S2: GSK3 deficiency dampens multi-organs and alters gut microbiota. Figure S3: Deletion efficiency of β-catenin in iDKO and iTKO mice. Figure S4: Original images with markers from western blot assay.
