# Supplementary material for: Glycogen Synthase Kinase 3 Is Essential for Intestinal Cell Niche and Digestive Function
Source: Biology (Basel). 2025 Nov 5;14(11):1551. doi: 10.3390/biology14111551 (PMC12650756; doi:10.3390/biology14111551)
Supplement: Supplementary file 1 [file biology-14-01551-s001.zip › biology-3929776-supplementary.pdf]

Article

Glycogen synthase kinase 3 is essential for intestinal cell niche and digestive function

Minggang Yang [1,2†], Xiaohui Li [3†], Jiajia Zhan [1], Rui Pan [1], Ziye Yang [1], Mengsha Zhou [2], Lei Ma [4*], Chenfeng Liu [1,2*]

[1] School of Life Science, Anhui Medical University, Hefei 230032, China; yang_mg@qq.com (M.Y.) 812198480@qq.com (J.Z.), 2820454677@qq.com (R.P.), ahmuziye@163.com (Z.Y)

[2] State Key Laboratory of Cellular Stress Biology, School of Life Sciences, Faculty of Medicine and Life Sciences, Xiamen University, Xiamen 361102, China. zhoumsh28@163.com (M.Z.)

[3] Department of Geriatrics, The 960Th Hospital of PLA, Jinan 250031, Shandong, China. xiaohuili070801@163.com (X.L)

[4] Tianjin Blood Center,Tianjin 300110，China.

* Correspondence: malei2186@163.com (L.M.); chenfengliu0607@163.com (C.L.)

† These authors contributed equally to this work.

Table S1. Primers for RT-qPCR

**Primer for qPCR**		
Lgr5	Forward	AGGGAACCGAGCCTTACAGA
	Reverse	TTCAAGGTCCCGCTCATCTTG
Dclk1	Forward	ATGTGGACCAGAGAAGTTCCG
	Reverse	CCGCCATGCTGAGAGATCC
Muc2	Forward	AGGGCTCGGAACTCCAGAAA
	Reverse	CCAGGGAATCGGTAGACATCG
ChgA	Forward	TACCCAATCACCAACCAGCC
	Reverse	GAGTCCGACTGACCATCATCT
DLL1	Forward	CCCATCCGATTCCCCTTCG
	Reverse	GGTTTTCTGTTGCGAGGTCATC
Alpi	Forward	CCACTCCCATGTCTTCGCAT
	Reverse	AAATATGGCCACGTCCTCCC
Lyz1	Forward	TGTCATGAGGCATTCAGGAGG
	Reverse	TCCTGTGGTTATTGGCTGGT
EphB2	Forward	TGTTTACAGCGATTCCGTGG
	Reverse	CCCTGTGACAGTTGGCTTTG
EphB3	Forward	CCTGTGCAGGTCTGACACTC
	Reverse	TGAAGAGGTTTGGGGCACAC
EphrinB1	Forward	CCAGGAAATCCGCTTCACCA
	Reverse	GGTAGTCAACTGCTCGGGTG
Nestin	Forward	GGTTGCGTCGGGGAAGAATC
	Reverse	CCAGCTCTTCGGCAAGGTT
Sst	Forward	GAGGTCTGCCAACTCGAACC
	Reverse	TCAGAGGTCTGGCTAGGACA
VGLUT2	Forward	TCTGTCCGTGGTCCTGAAATG
	Reverse	AACATGGTCTCCTCCCATCG
Elval4	Forward	GATCAGGGATGCTAACCTGTATG
	Reverse	GGTGATGATGCGACCGTATT

Hand2	Forward	GGCGGAGATCAAGAAGACCG
	Reverse	AGAAGTCCTCAGAACGGAGC
Nos1	Forward	ACAACCCTGCCATCACTAGC
	Reverse	ACACAGCAGTTCGGATGAGG
ChAT	Forward	TTGTTGCTGCTCCCCTATCC
	Reverse	AGAGCTACCATGTGCACCAC
HPRT	Forward	TGAAGAGCTACTGTAATGATCAGTCA
	Reverse	AGCAAGCTTGCAACCTTAACCA

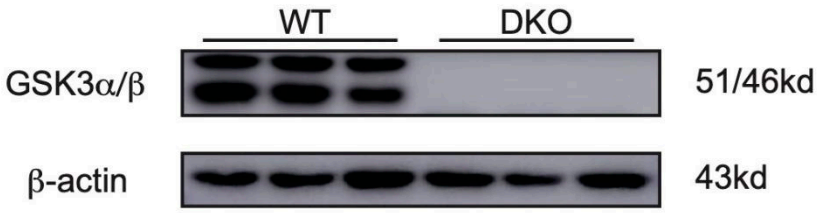

Figure S1: Deletion efficiency of GSK3α/β in DKO mice.

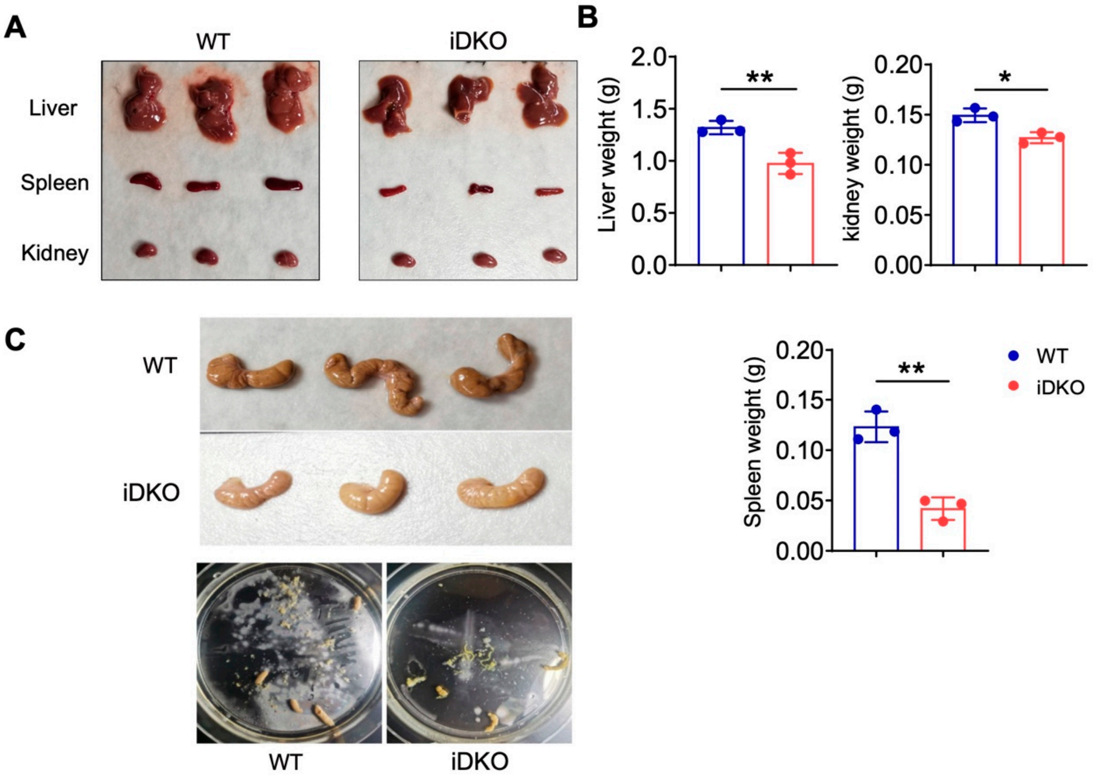

Figure S2: GSK3 deficiency dampens multi-organs and alters gut microbiota. WT and iDKO mice were used for comparative analysis of liver, spleen and kidney. Each group contained at least 3 mice, and all experiments were collected at least two independent experiment. (A) Representative images of the liver, kidney, and spleen from WT and iDKO mice. (B) Liver, kidney, and spleen weights in WT and iDKO mice (n=3). (C) There was a distinct color difference between the cecal content and the fecal pellets in WT and iDKO mice (n=3). Data are presented as mean ± SEM, and statistical significance: *, $p < 0.05$; **, $p < 0.01$.

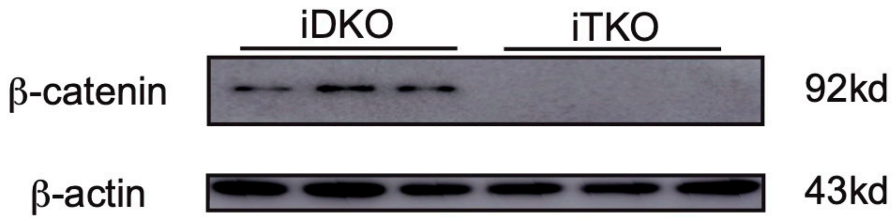

Figure S3: Deletion efficiency of β-catenin in iDKO and iTKO mice.

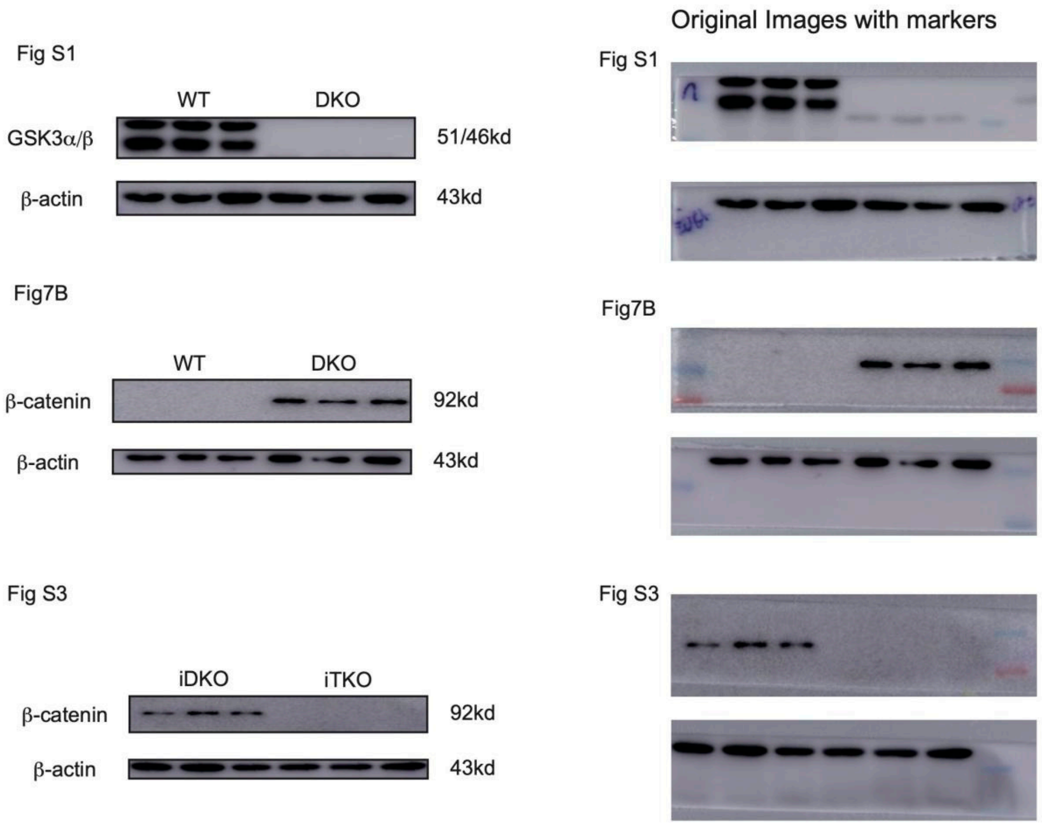

Figure S4：Original images with markers from western blot assay。
